# NMR and Docking Calculations Reveal Novel Atomistic Selectivity of a Synthetic High-Affinity Free Fatty Acid vs. Free Fatty Acids in Sudlow’s Drug Binding Sites in Human Serum Albumin

**DOI:** 10.3390/molecules28247991

**Published:** 2023-12-07

**Authors:** Themistoklis Venianakis, Alexandra Primikyri, Till Opatz, Stefan Petry, Georgios Papamokos, Ioannis P. Gerothanassis

**Affiliations:** 1 Section of Organic Chemistry and Biochemistry, Department of Chemistry, University of Ioannina, GR-45110 Ioannina, Greece; vethemis@gmail.com (T.V.); aleprimik@gmail.com (A.P.); 2 Department of Chemistry, Johannes Gutenberg-University, Duesbergweg, 10–14, 55128 Mainz, Germany; opatz@uni-mainz.de; 3 Sanofi-Aventis Deutschland GmbH, Integrated Drug Discovery, Industriepark Höchst, 65926 Frankfurt am Main, Germany; stefanmatthias.petry@sanofi.com; 4 Department of Physics, Harvard University, 17 Oxford Street, Cambridge, MA 02138, USA

**Keywords:** HSA, STD NMR, INPHARMA NMR, docking calculations, NBD-C_12_ FA

## Abstract

Saturation transfer difference (STD), inter-ligand NOEs (INPHARMA NMR), and docking calculations are reported for investigating specific binding sites of the high-affinity synthetic 7-nitrobenz-2-oxa-1,3-diazoyl-4-C_12_ fatty acid (NBD-C_12_ FA) with non-labeled human serum albumin (HSA) and in competition with the drugs warfarin and ibuprofen. A limited number of negative interligand NOEs between NBD-C_12_ FA and warfarin were interpreted in terms of a short-range allosteric competitive binding in the wide Sudlow’s binding site II (FA7) of NBD-C_12_ FA with Ser-202, Lys-199, and Trp-214 and warfarin with Arg-218 and Arg-222. In contrast, the significant number of interligand NOEs between NBD-C_12_ FA and ibuprofen were interpreted in terms of a competitive binding mode in Sudlow’s binding site I (FA3 and FA4) with Ser-342, Arg-348, Arg-485, Arg-410, and Tyr-411. NBD-C_12_ FA has the unique structural properties, compared to short-, medium-, and long-chain saturated and unsaturated natural free fatty acids, of interacting with well-defined structures with amino acids of both the internal and external polar anchor sites in Sudlow’s binding site I and with amino acids in both FA3 and FA4 in Sudlow’s binding site II. The NBD-C_12_ FA, therefore, interacts with novel structural characteristics in the drug binding sites I and II and can be regarded as a prototype molecule for drug development.

## 1. Introduction

**Figure 1:**
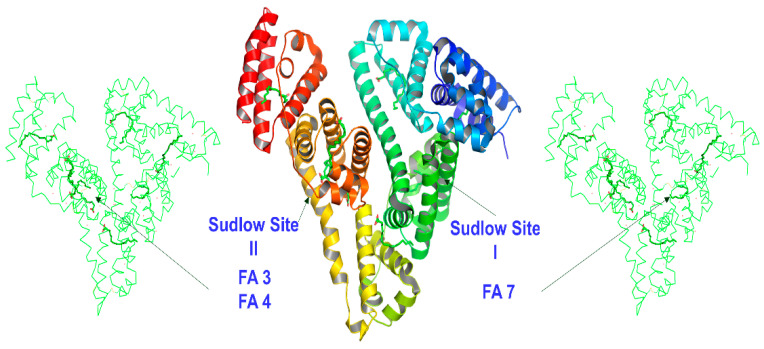
HSA Sudlow’s binding sites I and II. The fatty acid binding sites FA3/FA4 and FA7 are indicated on the left and right, respectively.

Human serum albumin (HSA) is the most abundant protein in blood plasma, with concentrations of 35–50 g/L and an average half-life of 19 days. It is very stable in a wide range of pH values (4 to 9), withstands temperatures up to 60 °C, and is highly soluble in various organic solvents such as 40% ethanol. HSA maintains oncotic pressure between the blood vessels and tissues; it binds bilirubin, the breakdown product of heme, and many therapeutic drugs such as indole compounds, benzodiazepines, sulfonamides, penicillins, etc. HSA transports a variety of fat-soluble hormones and numerous short-, medium-, and long-chain saturated, mono- and polyunsaturated free fatty acids (FFAs) to the liver and myocytes for energy utilization [1,2,3,4]. HSA is a monomeric globular protein of 585 amino acid residues with 17 disulfide bridges. It comprises a single nonglycosylated polypeptide chain with 67% α-helices without β-sheets. HSA contains three homologous helical domains, I, II, and III, divided into A and B subdomains, forming a heart-shaped molecule. Pioneering X-ray crystallography studies of Curry et al. [5,6,7,8] identified seven binding sites, denoted FA, for a variety of medium- and long-chain mono- and polyunsaturated FFAs. The sites FA1 and FA2 are in the subdomain IB and at the interface between subdomain IA and IIA, respectively. The sites FA3 and FA4 are in the subdomain III A and bind small-molecular-weight aromatic carboxylic acids, such as the drug ibuprofen (Sudlow’s drug binding site II [9], Figure 1). The FA5 is in the subdomain III B, and the FA6 is at the interface between IIA and IIB. The FA7 is in the subdomain IIA and binds heterocyclic negatively charged molecules, such as the drug warfarin (Sudlow’s drug binding site I [9], Figure 1). Two-dimensional ^1^H-^13^C HSQC experiments showed the presence of nine binding sites of ^13^C methyl-labeled oleic acid bound to HSA [10].

Competition of drugs with free fatty acids for human albumin Sudlow’s binding sites may significantly affect the potency of drugs [2,3,4,11,12]. Extensive X-ray structural data of a variety of short- and long-chain saturated, mono- and polyunsaturated FFAs in the binding site FA7 could be modeled only for the methylene trails without determination of the coordination of the carboxylate groups [5,6,7,8]. These data were interpreted regarding the low affinity of FFAs; thus, high concentrations of FFAs are required for the efficient displacement of the anticoagulant drug warfarin. Recent STD and INPHARMA NMR as well as docking calculations [13,14,15,16] provided a new aspect of molecular recognition of FFAs in FA7. The possibility of two entirely different binding modes of FFAs, due to the presence of two polar amino acid anchor sites, was concluded to be the main reason that the precise coordination of the carboxylic groups could not be obtained by X-ray crystallography.

Recently, a single high-affinity binding site was identified and characterized using the lipophilic derivative 7-nitrobenz-2-oxa-1,3-diazol-4-yl-C_12_ fatty acid (NBD-C_12_ FA) [17]. The structure of the HSA molecule is more similar to the fat-free structure (2.8 Å rmsd [18]) than the HSA structure with seven bound fatty acids (5.3 Å rmsd [6]). It was concluded, on the basis of X-ray and fluorescence experiments [17], that the binding site of the NBD-C_12_ FA conjugate is not identical with the warfarin binding site in HSA; however, it partly overlaps with the latter. A lower electron density near the side chain of Tyr-411, which is a critical amino acid residue for the binding of the drug ibuprofen, was also observed [17]. Fitting a second molecule of NBD-C_12_ FA resulted in strong electron density of the 4-nitrobenzoxadiazole group; however, the intensity of the fatty acid part of the molecule was very weak.

Understanding, at the atomic level, the selectivity of high-affinity ligands for HSA, such as the lipophilic fatty acid derivative NBD-C_12_ FA, is very important for drug discovery since they can compete effectively with free fatty acids for HSA Sudlow’s binding sites. We therefore report herein combined NMR (saturation transfer difference-STD) [13,14,15,16,19,20,21], 2D-Tr NOESY [22,23,24], and interligand NOEs for pharmacophore mapping (INPHARMA) [13,14,15,16,25,26,27]) as well as docking calculations [28,29,30,31] of the high-affinity ligand NBD-C_12_ FA in competition experiments with two drugs: warfarin, which is a stereotypical anticoagulant drug for FA7, and ibuprofen, which is an anti-inflammatory drug for FAs 3 and 4. A unified atomic level model for the selectivity of NBD-C_12_ FA vs. short-, medium-, and long-chain mono- and polyunsaturated FFAs is proposed.

## 2. Results and Discussion

### 2.1. STD and INPHARMA NMR Competition Experiments of NBD-C12 FA with Warfarin and Ibuprofen

#### 2.1.1. The FA7 Binding Site

**Figure 2:**
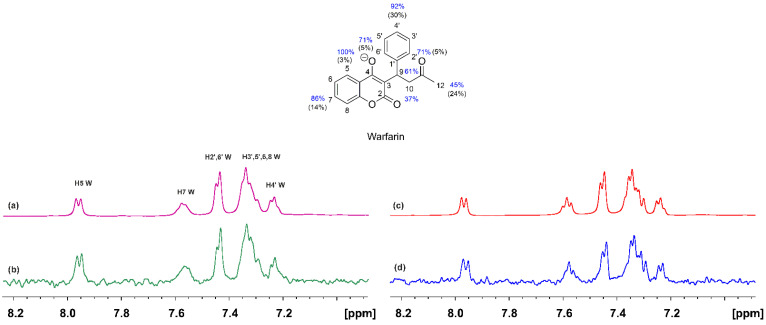
^1^H NMR spectra (500 MHz) of (**a**) warfarin (W) (2 mM) with native HSA (25 μΜ) in 50 mM PBS buffer in D_2_O with 20% DMSO-d_6_ (T = 323 K); (**c**) as in (**a**) after the addition of 2 mM of NBD-C_12_ FA; (**b**) STD ^1^H NMR spectrum of (**a**). (**d**) STD ^1^H NMR spectrum of (**c**). The STD amplification factor of warfarin in the binary HSA warfarin complex is shown in blue color and the % reduction upon addition of NBD-C_12_ FA is shown in black.

The ^1^H NMR spectrum of warfarin (W) (2 mM) with HSA (25 μM) is shown in Figure 2a. Despite the addition of an equimolar quantity of NBD-C_12_ (2 mM), the relative integrals of the H5(W) and H6′(NBD-C_12_) protons showed a molar ratio of W/NBD-C_12_~2/1, presumably due to very low solubility of the fatty acid analogue. Despite the low concentration of NBD-C_12_, a reduction in the linewidths of warfarin was observed, especially those of the aromatic H7 and the strongly overlapped H3′, 5′, 6, 8, and H4′. In addition, the linewidth of the H6′ and H5′ of NBD-C_12_ (Δν_1/2_ ≈ 20 Hz) is significantly larger than that of, e.g., the H5 of warfarin (doublet with Δν_1/2_ ≈ 5 Hz). Similar results were obtained with the STD NMR experiments. The STD NMR spectrum of warfarin in the presence of HSA shows strong resonances of the aromatic protons (Figure 2b). The epitope mapping of the protons of the bound warfarin was evaluated with the determination of the STD amplification factor (A_STD_), which reflects the proximity of the protons to the binding site on HSA. The STD signals were normalized with respect to the signal with the highest A_STD_ values, which was set to 100%. All the protons show A_STD_ values above 37%, which shows the efficient binding of warfarin to HSA. The STD line widths and intensities of warfarin bound to HSA upon adding NBD-C_12_ FA are reduced (Figure 2d), especially those of the H7, H3′, 5′, 6, 8, and H4′, with a reduction of the A_STD_ values in the range of 5 to 37% (Figure 2).

Extensive complexation studies of warfarin with HSA showed a wide range of formation constants (~1.4 × 10^6^–2.3 × 10^3^ M^−1^) depending on the experimental techniques and conditions used [32,33,34,35]. A formation constant of ~2 × 10^5^ M^−1^ was determined for warfarin by the switch sense method, which can be compared with the values for the NBD-C_12_ FA of ~0.37 × 10^5^ M^−1^ by fluorescence titration and the switch sense method and ~0.68 × 10^5^ M^−1^ by dialysis [17].

The above NMR competition experimental data can be analyzed in terms of: (i) two ligands which are competitive towards the FA7 binding site; (ii) short-range (<5 Å) allosteric interaction in the wide FA7 site; and (iii) long-range (>5 Å) allosteric interaction which results in conformational changes in FA7 and, thus, a decrease in the affinity of warfarin.

**Figure 3:**
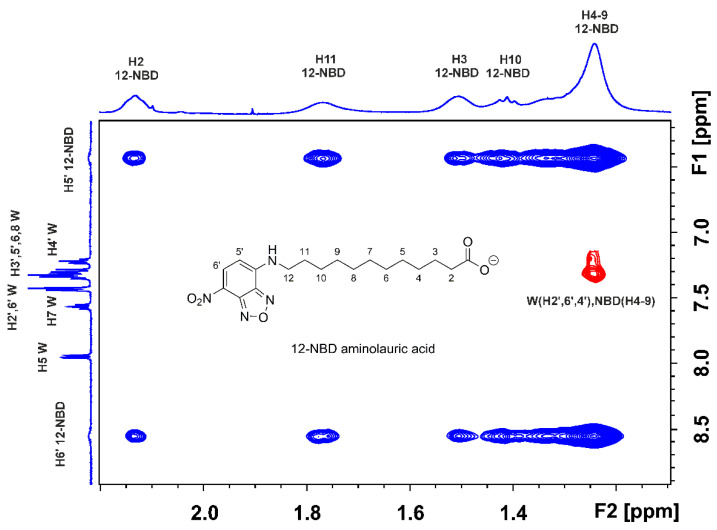
Interligand 2D Tr-NOESY (INPHARMA) NMR spectrum (500 MHz) of NBD-C_12_ FA (0.8 mM saturated solution) in the presence of warfarin (W) (1.6 mM) with native HSA (25 μΜ) in 50 mM PBS buffer in D_2_O with 20% DMSO-d_6_, T = 323 K, mixing time = 300 ms. The red cross-peaks denote interligand NOEs between NBD-C_12_ FA and warfarin.

The use of the 2D Tr-NOESY (INPHARMA) NMR technique [25,26], which is based on observing inter-NOEs between two ligands that bind competitively to a common binding site with distances < 5 Å, can be utilized to resolve the above ambiguity. The competition experiment of NBD-C_12_ FA with warfarin (Figure 3) shows the presence of a limited number of inter-NOEs (denoted with the red cross-peaks) between the H2′, 6′, and H4′ protons of the phenyl ring of warfarin and the H4-9 protons of NBD-C_12_ FA, which are close in space (<5 Å). Of particular interest is the absence of common inter-NOEs between the aromatic protons of the two ligands, which demonstrates that the phenyl butyl and benzopyran ring of warfarin and the 7-nitrobenz-2-oxa-1,3-diazol-4-yl moiety of NBD-C_12_ FA are at distances > 5 Å, i.e., beyond the detection limits of NOE experiments. Cross-peaks between the two ligands in the absence of HSA were not observed. This demonstrates that the interligand NOEs of Figure 3 result from a spin-diffusion process through the HSA protons due to the partial proximity of the phenyl group of warfarin and the 4–9 protons of NBD-C_12_ FA in the binding site FA7. Nevertheless, the presence of a limited number of inter NOE connectivities between NBD-C_12_ FA and warfarin is contrary to the significant number of negative 2D interligand NOEs that were observed between short (caproleic), medium (oleic, linoleic, and α-linolenic acids) and long (EPA and DHA) FFAs and warfarin [13,14,15,16], which demonstrates a common binding mode in FA7 and the presence of two polar amino acid anchor sites (see discussion on docking calculations).

#### 2.1.2. The FA3 and FA4 Binding Sites

**Figure 4:**
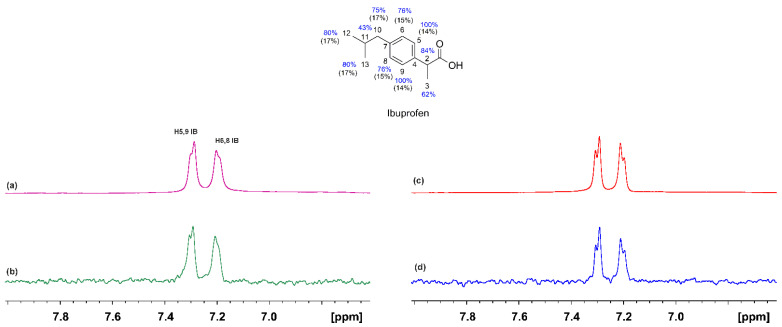
^1^H NMR spectra (500 MHz) of: (**a**) ibuprofen (I) (2 mM) with native HSA (25 μΜ) in 50 mM PBS buffer in D_2_O with 20% DMSO-d_6_ (T = 323 K); (**c**) as in (**a**) after the addition of 2 mM of NBD-C_12_ FA; (**b**) STD ^1^H NMR spectrum of (**a**). (**d**) STD ^1^H NMR spectrum of (**c**). The STD amplification factor of ibuprofen in the binary HSA ibuprofen complex is shown in blue color and the % reduction upon the addition of NBD-C_12_ FA is shown in black.

The ^1^H NMR spectrum of ibuprofen in complexation with HSA is shown in Figure 4a. Despite the addition of NBD-C_12_ FA at a molar ratio of 1/1, the resulting relative integrals of the H5,9 (IB) and H6′ (NBD-C_12_ FA) showed a molar ratio of IB/NBD-C_12_ FA~2/1 to 4/1 due to low solubility of the synthetic analogue. Despite the significantly smaller concentration of NBD-C_12_ FA, a reduction in the linewidth of ibuprofen is observed (Figure 4c), which probably reflects competition towards the same binding site. A similar conclusion can be drawn from the STD experiments (Figure 4b,d). Again, the epitope mapping of the protons of the bound ibuprofen was evaluated with the determination of the STD amplification factor (A_STD_). All the protons show A_STD_ values above 62%, which shows the efficient binding of ibuprofen with HSA. Addition of NBD-C_12_ FA shows a reduction of the A_STD_ values in the range of 14 to 17%. Nevertheless, to assess whether the reduction in the line widths and amplitude of the STD signals reflects competitive interactions towards the FA3 and FA4 binding sites or, rather, that NBD-C_12_ FA binds at a different site and results in long-range conformational changes in FA3/FA4 (long-range allosteric inhibition), the 2D-Tr NOESY (INPHARMA) NMR method was applied.

**Figure 5:**
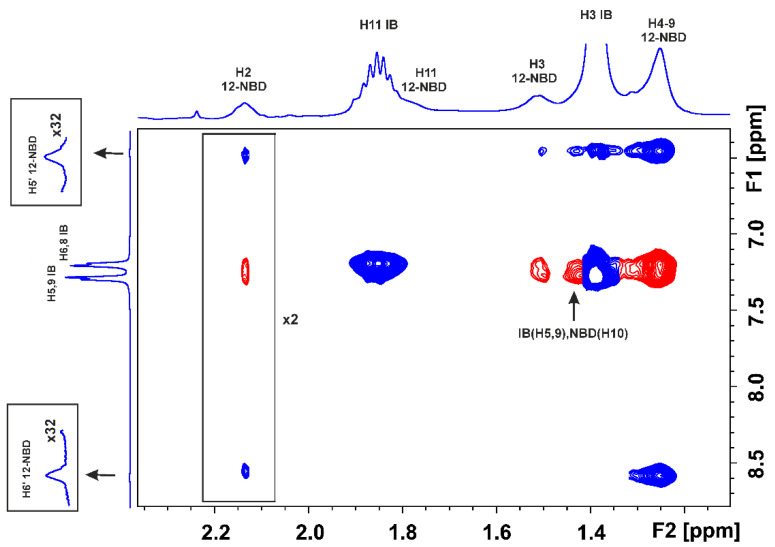
Interligand 2D Tr-NOESY (INPHARMA) NMR spectrum (500 MHz) of NBD-C_12_ FA (0.8 mM saturated solution) in the presence of ibuprofen (IB) (1.6 mM) with native HSA (25 μΜ) in 50 mM PBS buffer in D_2_O with 20% DMSO-d_6_, T = 323 K, mixing time = 300 ms. Interligand NOEs between NBD-C_12_ FA and ibuprofen are denoted with the red cross-peaks (x is a multiplication factor).

Figure 5 shows the 2D Tr-NOESY (INPHARMA) NMR competition experiment of NBD-C_12_ FA and ibuprofen which indicates the presence of very characteristic negative inter-NOEs (denoted with the red cross-peaks; in-phase with respect to those of the diagonal) between H5,9 and H6,8 of ibuprofen with the H2, H3, and H4–9 of NBD-C_12_ FA. Significant interligand NOE connectivities were also observed between H2 of ibuprofen with H3 and H2 of NBD-C_12_ FA. Similar results were obtained with 2D Tr-NOESY (INHPARMA) NMR competition experiments of NBD-C_12_ FA (400 μM) and ibuprofen (400 μM) using mixing times of 300 ms and 200 ms (Figure S1). This finding confirms NOE transfer between the two ligands with distances < 5Å and, thus, competition towards a common binding site. Cross-peaks between the two ligands in the absence of HSA were not observed (Figure S2), which demonstrates that the interligand NOEs of Figure 5 are not due to the direct transfer of magnetization between ibuprofen and NBD-C_12_ FA; they are mediated from a spin-diffusion process through the HSA protons.

### 2.2. Docking Calculations

Molecular docking is a valuable method in drug design. Thus, millions of molecules of known structures retrieved from virtual libraries are tested against drug targets using high-performance computers and high-scalability software tools [36]. Nonetheless, several deficiencies have been reported in the past [37,38], and various solutions have been proposed to overcome the limits of the accuracy of this method [39,40]. Recent developments include newly established techniques such as deep learning [41] that may address the known problem of irreproducibility in biomedical research [42].

In our previous studies, we have used an approach based on site-specific docking, guided by experimental results of NMR and X-ray crystallography, which has proven very successful in locating poses consistent with experimental results [13,15,27]. The question set by the experimental results of NMR and the X-ray crystallography is the structural arrangement of the drugs warfarin (W) at the binding site FA7 and ibuprofen (IB) at binding sites FA3 and FA4. The preferred structural arrangement, according to the experimental results generated by the above techniques, must fulfill the following spatial requirements:

1. The (7-nitrobenz-2-oxa-1,3-diazol-4-yl)-C_12_ fatty acid [17] and the drug warfarin interact weakly through the FA’s methylene groups and the drug’s phenyl butyl moiety in the binding site FA7.
2. The NBD-C_12_ fatty acid interacts with the drug ibuprofen in the binding sites FA3 and FA4.

#### 2.2.1. The FA7 Binding Site

**Figure 6:**
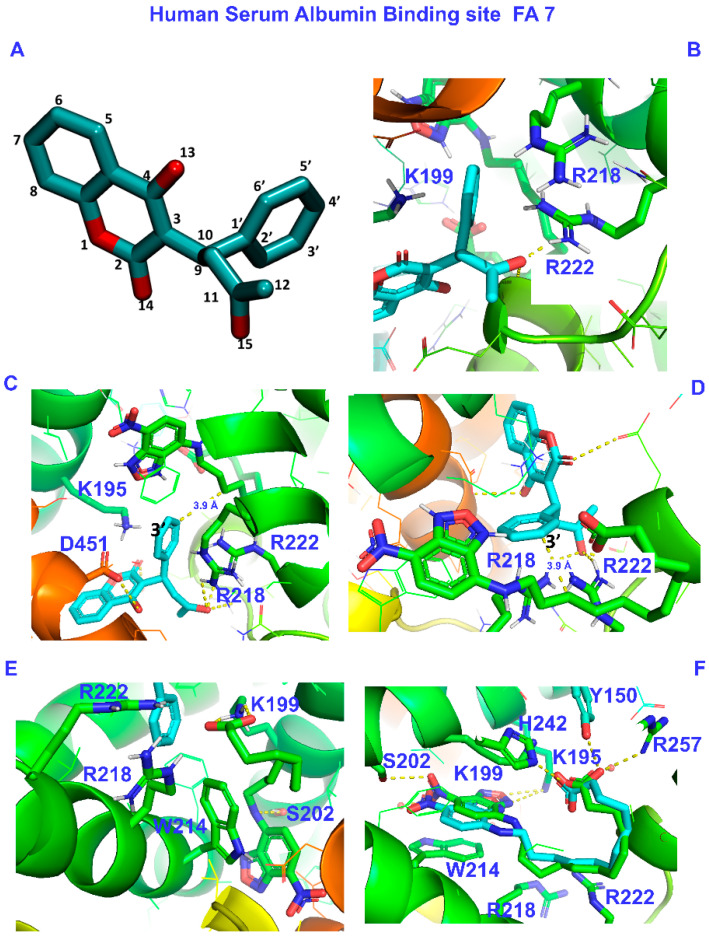
(**A**) Atom-numbering of warfarin molecule. (**B**–**D**). Different views of successful docking pose of warfarin in the presence of NBD-C_12_-FA. (**E**) Successful docking pose of NBD-C_12_-FA in the presence of warfarin. (**F**) Self-docking of NBD-C_12_-FA and superposition with the crystal structure.

The FA7 binding site, although primarily hydrophobic, contains two clusters of polar amino acids: an internal one with amino acids Tyr-150, His-242, and Arg-257 and an external one, in the entrance of the pocket, with amino acids Lys-195, His-242, Arg-218, and Arg-222 [8,43]. Initially, we performed site-specific warfarin docking in the presence of NBD-C_12_ FA (pdb code: 6ezq.pdb). To be consistent with the above NMR results, we varied the grid and the box’s center accordingly and selected the poses that agreed with the experiment. The docking results indicate that warfarin interacts with amino acids Arg-218 and Arg-222 of the external cluster. The binding also includes Lys-195 and Asp-451. The docking calculations showed that carbon No 10 (C10) of the NBD-C_12_ FA is close to the single aromatic ring (3.9 Å). These results are presented in Table 1 and Figure 6A–D.

In a subsequent step, we repeated the site-specific docking (FA7), for NBD-C_12_ FA, in the presence of warfarin (crystal structure pdb code: 2BXD.pdb). The results are shown in Table 1 and Figure 6E. The carboxylate group interacts with Lys 199, Arg-218, and Arg-222 of the external cluster. The nitro group terminal side interacts with Ser-202 through the NH group, and there is a pi-pi stacking interaction between the aromatic rings of NBD and Trp-214.

Additionally, we performed site-specific docking to verify the accuracy of our approach. Consequently, we removed NBD-C_12_ FA from its crystal structure (6EZQ.pdb) and used the free protein and NBD-C_12_ FA as ligands (self-docking). The binding, depicted by X-ray, was reproduced with our docking calculations: The nitro group interacts with Ser-202 and Trp-214, and the carboxylate group at FA7 interacts with Lys-199 and His-242 The docking calculations revealed that Tyr-150, Lys-195, and Arg-257 may also be involved in the binding (see Figure 6F below and Figure 3A in reference [17]).

From the results of this work, it is evident that warfarin occupies the remaining space of FA7 left free by the NBD-C_12_ FA, which is in line with previous observations from fluorescence titration experiments [17]. Although the interaction of warfarin with residues Arg-218 and Arg-222 is expected from X-ray [8] and our docking calculations [13], the Lys-195 residue seems to alter the interaction in the FA7.

The crystal structure of HSA-NBD-C_12_ FA (6EZQ) shows that the nitro group interacts with Ser-202 and Trp-214, and the carboxylate group interacts with Lys-199 and His-242 (see also Figure 3A in reference [17]). Moreover, upon binding of warfarin, a spatial rearrangement is observed for HSA, which has also been noticed before [9,44]. The authors showed that warfarin and NBD-C_12_ FA binding does not constitute a competitive mechanism [17]. Our docking results, which meet the demands of the crystal structure and NMR findings, indicate a non-competitive (allosteric) binding of warfarin at FA7, which can also alter this binding site. Additionally, to account for the fluorescence results [17], we performed flexible docking calculations of warfarin in the presence of the NBD-C_12_ FA ligand. The resulting orientations of the flexible aromatic rings of NBD-C_12_ FA and Trp-214 remained essentially the same. A slight change in the relative arrangement of the NBD moiety and Trp-214 or the repulsion of a molecule of water from the cavity can result in an increased NBD fluorescence.

#### 2.2.2. The FA3 and FA4 Binding Sites

Our docking results show that the NBD-C_12_ FA occupies the FA3 entirely through interactions of the carboxylate group with Ser-342, Arg-348, and Arg-485. The nitro group interacts with Arg-410 and Tyr-411 in one of the anchoring sites of FA4 (cluster 1, Table 2), which is more elongated and narrow than the site FA3. These interactions are similar to those based on the X-ray electron density and on fluorescence data [17]. A binding site of ibuprofen in FA3 was previously identified by docking calculations [13] which also involve Ser-342, Arg-348, and Arg-485 (Table 3). This is in agreement with the common interligand NOE connectivities of H2 of ibuprofen with H3 and H2 of NBD-C_12_ FA ligand.

**Figure 7:**
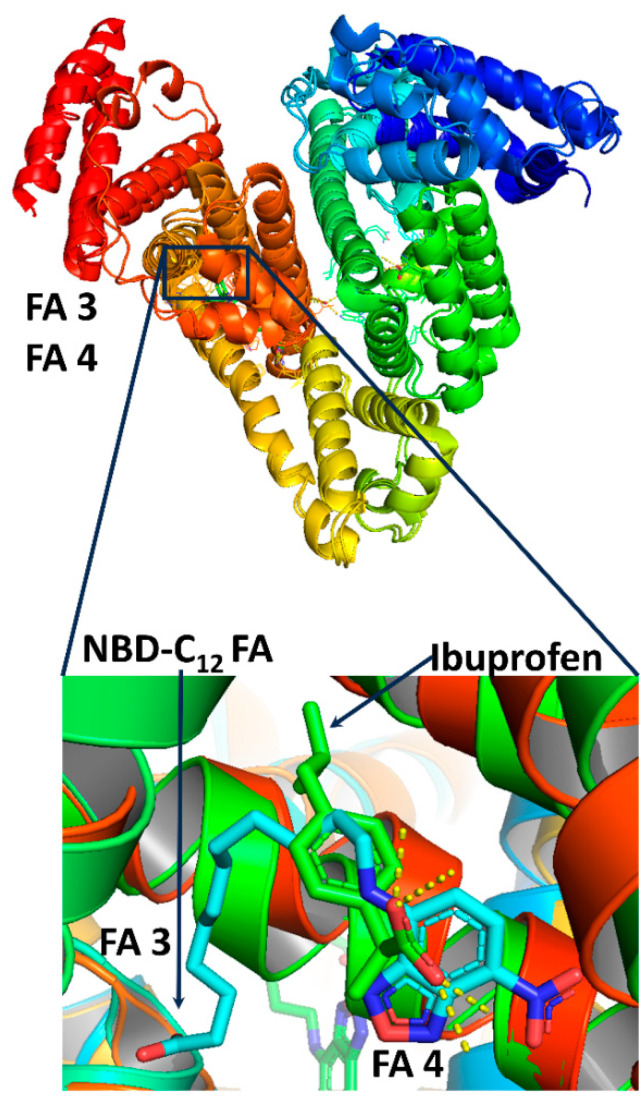
Superposition of NBD-C_12_ FA and ibuprofen in FA3/FA4 binding sites (crystal structure codes in PBB: 6ezq.pdb and 2bxg.pdb).

Repeated docking simulations of ibuprofen in the presence of NBD-C_12_ FA systematically failed to bind in FA3 since the carboxyl group of the NBD-C_12_ FA does not allow such interaction. Ibuprofen binding is possible only when this interacts with the anchoring site comprised of Arg-410 and Ser-411, which is identical to NBD-C_12_ FA. Docking calculations for ibuprofen in FA4 indicate that interaction occurs out of the protein cage, entailing a lack of binding. Based on our NMR experiments and the conclusion of Wenskowsky et al. [17] that ibuprofen shows no appreciable effect at the relatively low concentrations in their fluorescence assay, we conclude that (a) for the NBD-C_12_ FA, the FA7 is the primary binding site and (b) the significantly higher concentration of ibuprofen relative to that of NBD-C_12_ FA resulted in observable antagonistic phenomena in our STD and 2D Tr-NOESY experiments. Figure 7 shows a superposition of NBD-C_12_ FA and ibuprofen in FA3/FA4 binding sites, in excellent agreement with our 2D Tr-NOESY experiments.

### 2.3. A Unified Atomic Level for the Selectivity of NBD-C_12_ FA and Short, Medium, and Long Mono- and Polyunsaturated Free Fatty Acids

We have recently demonstrated the presence of two orientations of mono- and polyunsaturated FFAs in the warfarin binding site FA7 due to the presence of two potential polar anchor sites: an inner cluster composed of the amino acids Tyr-150, His-242, and Arg-257 and an external cluster of the amino acids Lys-195, Lys-199, Arg-218, and Arg-222 [13,15,16]. Interestingly, increasing the length and polyunsaturation of the chain increases the affinity of FFAs due to hydrophobic interactions and the ability to adopt folded conformations [15]. The NBD-C_12_ FA binding mode in FA7 in the presence of warfarin is unique since the carboxylate group binds to amino acids in the external cluster (Lys-199, Arg-218, and Arg-222, Table 2). The NO_2_ group binds to amino acids in the inner cluster (Ser-202 and Trp-214, Table 2), which significantly reorganizes this binding site. Binding of NBD-C_12_ FA, therefore, modifies the FA7 binding site without changing the overall structure. The significant remaining space allows the binding of warfarin in the external cluster with limited contacts with the NBD-C_12_ FA molecule.

The binding mode of the carboxylate group of NBD-C_12_ FA in FA3 is identical to that of DHA and EPA and involves interactions with Ser-342, Arg-348, and Arg-485 (Table 3). The NO_2_ group interacts with Arg-410 and Tyr-411 of cluster 1 in FA4, which results in significant high affinity. The remaining space of cluster 2 of FA4 is insufficient for the accommodation and interaction with ibuprofen.

## 3. Material and Methods

### 3.1. Chemicals and Reagents

Warfarin and ibuprofen, purity ≥ 98% (GC), and human serum albumin fatty acid–depleted lyophilized powder, purity ≥ 96% (agarose gel electrophoresis), were obtained from Sigma Aldrich Chemie, GmbH, Taufkirchen, Germany. D_2_O and DMSO-d_6_ (>99.8%) were obtained from Deutero GmbH, Kastellaun, Germany. The NBD-C_12_ FA was synthesized according to [17].

### 3.2. NMR Experiments

STD and 2D Tr-NOESY (INPHARMA) NMR experiments were performed on a Bruker AV-NEO-500 spectrometer in the presence of HSA (25 μM) and 50 mM PBS (pD = 7.4) in D_2_O with 20% DMSO-d_6_ at 323 K to facilitate the dissolution of NBD-C_12_ FA. STD experiments were performed with selective saturation of 2 s with a train of Gaussian-shaped pulses, as previously reported [15,16,27]. Two-dimensional Tr-NOESY (INPHARMA) experiments were recorded with 80 scans, 2 K data block with 110 incremental values of the revolution times, and total experiment time ~15 h.

### 3.3. Computational Methods

Details of the computational approach are discussed in Refs [13,15,39]; herein, a summary is provided. The crystal structure of the complex of human serum albumin (HSA) with the ligand (7-nitrobenz-2-oxa-1,3-diazol-4-yl)-C_12_ (NBD-C_12_) FA was obtained from the Protein Data Bank. The entry code name is 6EZQ. Since experimental data indicate the coexistence of NBD-C_12_ FA and warfarin or ibuprofen at the binding sites 3–4 and 7, we did not remove the NBD-C_12_ FA from the initial structure. In contrast, we attempted site-specific docking with NBD-C_12_ present and defining a search space consistent with the known amino acids responsible for the binding of warfarin and ibuprofen, excluding those that the NBD-C_12_ FA occupies. The AutoDock Vina1.1.2 [30] software package was employed for the docking calculations. The AutoDock Tools 1.5.6 software package [29] was used as a preprocessing software package to add hydrogen atoms to the protein and select the search space for each complex studied. The selection of the poses was based on (a) inter-residue NOE intensities in competition experiments, (b) the highest affinity (10 independent runs for each binding site), and (c) minimum deviation from the X-ray crystal structure. Configuration files of docking are provided in Supplementary Materials.

## 4. Conclusions

The present work highlights the great potential of the combined use of 2D Tr-NOESY (INPHARMA) NMR and computational methods [13,15,16,26,27,45] to investigate structural and functional aspects of ligand–macromolecule interactions. More specifically:

- The limited number of negative interligand NOEs between H4–9 protons of NBD-C_12_ FA and protons of the phenyl ring of warfarin and the absence of common inter-NOEs between the aromatic rings of the two ligands were interpreted in terms of a short-range negative allosteric competitive binding of NBD-C_12_ FA with the amino acids Ser-202, Lys-199, Trp-214, and warfarin with Arg-218 and Tyr-411 in the wide binding site FA7.
- The extensive number of interligand NOEs between H2, H3, and H4–9 of NBD-C_12_ FA and the aromatic protons H5,9 and H6,8 of ibuprofen was interpreted in terms of a competitive binding mode with Ser-342, Arg-348, Arg-485, Arg-410, and Tyr-411 in the binding sites FA3 and FA4.
- The self-docking protocol of the ligands NBD-C_12_ FA, warfarin, and ibuprofen on the X-ray HSA–ligand structure allowed us to define the search space as precisely as possible and, thus, accurately define electrostatic and hydrogen bond interactions between ligands and HSA.
- Compared to short-, medium-, and long-chain mono- and polyunsaturated FFAs, the NBD-C_12_ FA has the unique structural characteristics of interacting with amino acids of both the internal and external clusters in Sudlow’s binding site I. In Sudlow’s binding site II, the NBD-C_12_ FA interacts with amino acids in both FA3 and FA4.
- X-ray and NMR-based docking calculations with site-specific docking has been proven to constitute a very successful method to elucidate and describe the generated electrostatic and H-bonded interactions between the ligands and the HSA protein at an atomic level.

The NBD-C_12_ FA, therefore, results in a significant reorganization in Sudlow’s drug binding sites and, thus, could be important for drug depot development and improved pharmacokinetics. Further studies are currently underway to investigate polyunsaturated FFAs conjugates with drugs to further understand drug–HSA binding modes.

## Figures and Tables

**Table 1:** Results from docking calculations. Electrostatic and hydrogen bond interactions in the binding site FA7 of HSA, successful pose, and affinities in kcal/mol. ^1^. Docking of warfarin in the presence of NBD-C_12_ FA (crystal structure employed: 6EZQ.pdb) ^2^. Docking of NBD-C_12_ FA in the presence of warfarin (crystal structure: 2BXD.pdb).

	HSA/Amino Acid Group	Dist. (Å)	Pose /Predicted Affinity (kcal/mol)
1. Atom-No of Warfarin			
-	K-199		6/−5.5
O_15_	R-218/NH_2_ η ^1^	3.0	
O_15_	R-222/NH_2_ η ^2^	3.1	
O_13_	D-451/OD2	3.3	
C_4_′	K-195/N ζ	3.6	
C_3_′	NBD/C_10_	3.9	
2. Group or Atom-No of NBD-C_12_ FA			
COO^−^	K-199	2.1	6/−6.5
COO^−^	R-222	3.4	
COO^−^	R-218	4.0	
Aromatic ring-C_2_′	W-214	3.5	
NH	S-202	1.9	

**Table 2:** Comparison of electrostatic and hydrogen bond interactions of DHA, EPA, ALA, NBD-C12 FA, and warfarin in the two anchor sites of FA7.

FA7		
Ligand	Inner Cluster	External Cluster
	Tyr-150, His-242, Arg-257	Lys-195, Lys-199, Arg-218, Arg-222
DHA (docking) ^a^	His-242, Arg-257 (−7.0 kcal/mol)	Lys-199, Arg-218, Arg-222 (−7.0 kcal/mol)
EPA (docking) ^a^	His-242, Arg-257 (−6.7 kcal/mol)	Lys-199, Arg-218, Arg-222 (−6.8 kcal/mol)
Warfarin (docking) ^b^	His-242, Arg-257 (−7.0 kcal/mol)	Arg-218, Arg-222 (−7.7 kcal/mol)
NBD-C_12_ (X-ray)	His-242, Ser-202 NO_2_	Lys-199, Trp-214 COO^−^
NBD-C_12_ (docking)	His-242, Ser-202 NO_2_	Lys-199, Trp-214 COO^−^
	(−7.3 kcal/mol)	
Warfarin in the presence of NBD-C_12_ (docking)	-	Lys-195, Arg-218, Arg-222
	(−5.5 kcal/mol)	
NBD-C_12_ in the presence of warfarin (docking)	Ser-202, Trp-214	Lys-199, Arg-218, Arg-222
	NO_2_	COO^−^
	(−6.5 kcal/mol)	

^a^ Ref. [15], ^b^ Ref. [13].

**Table 3:** Comparison of electrostatic and hydrogen bond interactions of ALA, DHA, NBD-C_12_ FA, and ibuprofen in the anchor sites of FA3 and FA4.

	FA3	FA4	
		Cluster-1	Cluster-2
DHA (docking) ^a^	Ser-342, Arg-348, Arg-485 (−8.3 kcal/mol)	Arg-410, Tyr-411 (−7.5 kcal/mol)	Ser-419, Thr-422 (−7.8 kcal/mol)
EPA (docking) ^a^	Ser-342, Arg-348, Arg-485 (−7.9 kcal/mol)	Arg-410, Tyr-411 (−7.0 kcal/mol)	Ser-419, Thr-422 (−7.8 kcal/mol)
Ibuprofen (docking) ^b^	Ser-342, Arg-348, Arg-485 (−7.2 kcal/mol)	Arg-410, Tyr-411 (−7.3 kcal/mol)	
Ibuprofen (X-ray)		Arg-410, Tyr-411	
NBD-C_12_ (X-ray)	Ser-342, Arg-348, Arg-485 COO^−^	Arg-410, Tyr-411 NO_2_	
NBD-C_12_ (docking)	Ser-342, Arg-348, Arg-485 COO^−^	Arg-410, Tyr-411 NO_2_	
	(−8.3 kcal/mol)		
Ibuprofen in the presence of NBD-C_12_ (docking)	-	-	

^a^ Ref. [15], ^b^ Ref. [13].

## Data Availability

Data are contained within the article and Supplementary Materials.

## Supplementary Materials

The following supporting information can be downloaded at https://www.mdpi.com/article/10.3390/molecules28247991/s1: Figure S1: Interligand 2D Tr-NOESY (INPHARMA) NMR spectrum (500 MHz) of NBD-C_12_ FA (400 μΜ) in the presence of ibuprofen (I) (400 μΜ) with native HSA (25 μΜ) in 50 mM PBS buffer in D_2_O with 20% DMSO-d_6_, T = 323 K, mixing time = 200 ms. Interligand NOEs between NBD-C_12_ FA and ibuprofen are denoted with the red cross-peaks. Figure S2: Interligand 2D Tr-NOESY NMR spectrum of NBD-C_12_ FA (0.8 mM, saturated solution) and ibuprofen (IB) (1.6 mM) in 50 mM PBS buffer in D_2_O with 20% DMSO-d_6_, T = 323 K, mixing time = 300 ms. The H6′ and H5′ cross-peaks of NBD-C_12_ FA and H5,9 and H6,8 of ibuprofen are anti-phase with respect to the diagonal due to fast molecular tumbling of the ligands within the extreme narrowing condition. Configuration files of docking calculations: warfarin–HSA docking in the presence of NBD-C_12_; NBD-C_12_–HSA docking in the presence of warfarin; NBD-C_12_–HSA docking in free HSA.
