# Supplementary material for: NMR and Docking Calculations Reveal Novel Atomistic Selectivity of a Synthetic High-Affinity Free Fatty Acid vs. Free Fatty Acids in Sudlow’s Drug Binding Sites in Human Serum Albumin
Source: Molecules. 2023 Dec 7;28(24):7991. doi: 10.3390/molecules28247991 (PMC10745614; doi:10.3390/molecules28247991)
Supplement: Supplementary file 1 [file molecules-28-07991-s001.zip › molecules-2727043-supplementary.pdf]

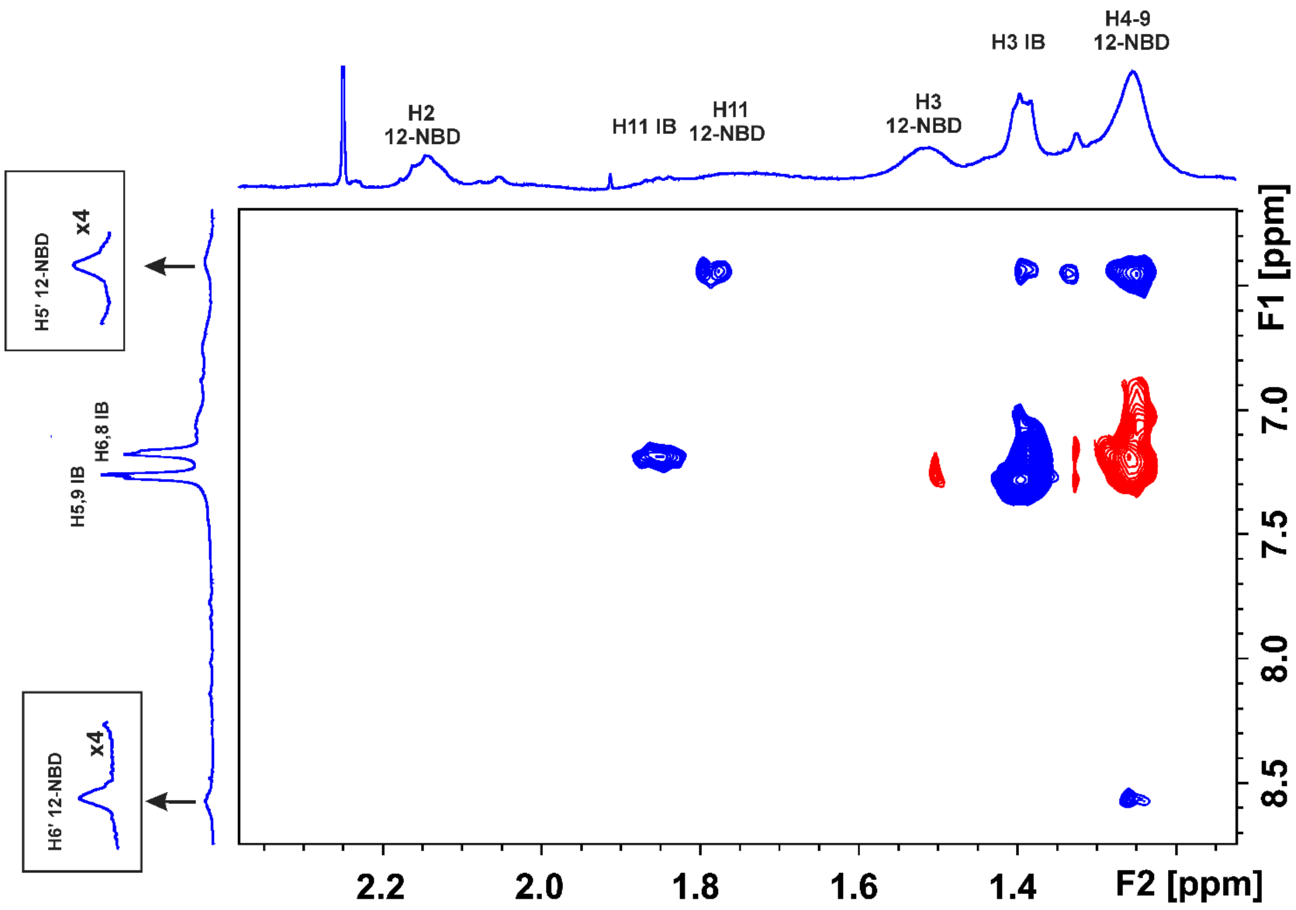

Figure S1. Inter-ligand 2D Tr-NOESY (INPHARMA) NMR spectrum (500 MHz) of NBD-C_{12} FA (0.4 mM) in the presence of ibuprofen (IB) (0.4 mM) with native HSA (25 µM) in 50 mM PBS buffer in D_2O with 20% DMSO-d_6, T = 323 K, mixing time = 200 ms. Inter-ligand NOEs between NBD-C_{12} FA and ibuprofen are denoted with the red cross-peaks.

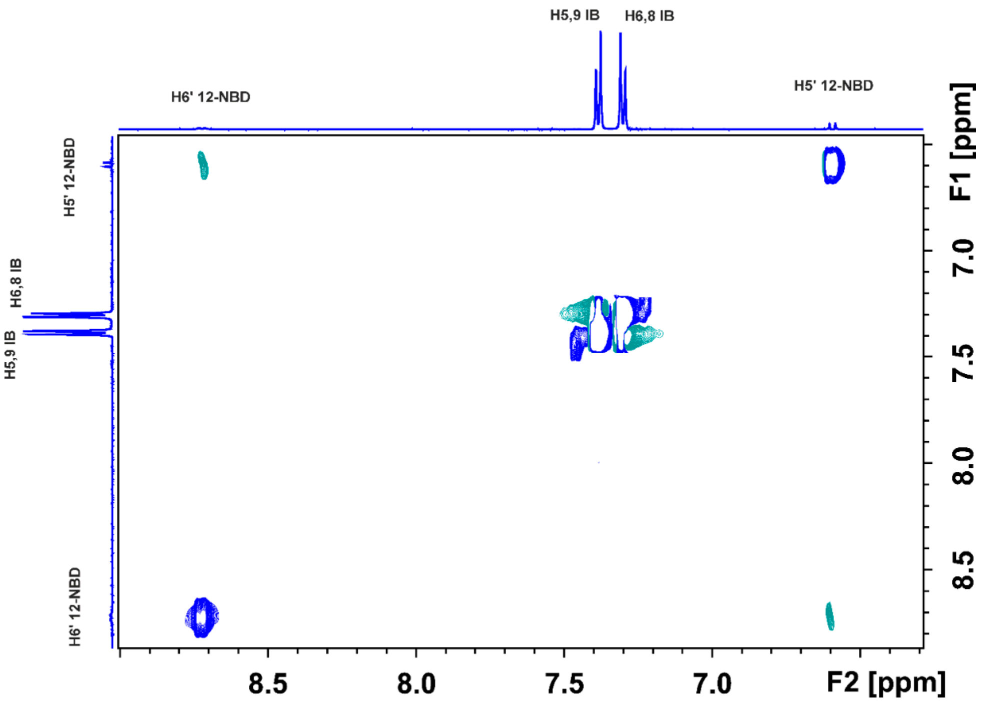

Figure S2. Inter-ligand 2D Tr-NOESY NMR spectrum of NBD-C12 FA (0.8 mM, saturated solution) and ibuprofen (IB) (1.6 mM) in 50 mM PBS buffer in D2O with 20% DMSO-d6, T = 323 K, mixing time = 300 ms. The H6′ and H5′ cross-peaks of NBD-C12 FA and H5,9 and H6,8 of ibuprofen are anti-phase with respect to the diagonal due to fast molecular tumbling of the ligands within the extreme narrowing condition.

Configuration file for warfarin–HSA docking in the presence of NBD-C_{12}

receptor = 6n.pdbqt (= 6ezq. pdb)
ligand = warfarin_i.pdbqt
center_x = -24.355
center_y = 12.645
center_z = 25.75
size_x = 22
size_y = 20
size_z = 20
exhaustiveness = 20

Configuration file for NBD-C_{12}–HSA docking in the presence of warfarin

receptor = hsaw.pdbqt (= 2bxd.pdb)
ligand = nbd.pdbqt
center_x = 40.973
center_y = 31.186
center_z = 58.409
size_x = 18
size_y = 12
size_z = 20
exhaustiveness = 20

Configuration file for NBD-C_{12}–HSA docking in free HSA

receptor = 6ezqf.pdbqt = (6ezq.pdb without NBD)
ligand = nbd.pdbqt
center_x = -25.783
center_y = 17.517
center_z = 29.83
size_x = 18
size_y = 18
size_z = 18
exhaustiveness = 20
